# Evaluation of Electrocardiographic T-peak to T-end Interval in Subjects with Increased Epicardial Fat Tissue Thickness

**DOI:** 10.5935/abc.20150124

**Published:** 2015-12

**Authors:** Ozgur Kaplan, Ertugrul Kurtoglu, Gokay Nar, Erdogan Yasar, Gokhan Gozubuyuk, Cem Dogan, Ahmet Ugur Boz, Sıho Hidayet, Hasan Pekdemir

**Affiliations:** 1 Department of Cardiology - İstanbul Bilim University School of Medicine, İstanbul, Turkey; 2 Department of Cardiology - Malatya State Hospital, Malatya, Turkey; 3 Department of Cardiology - Ahi Evran University Education And Research Hospital, Kırsehir,Turkey; 4 Department of Cardiology - İnönü University School of Medicine, Malatya, Turkey

**Keywords:** Pericardium, Adipose Tissue, Electracardiography, Arrhythmias, Cardiac, Reference Values

## Abstract

**Background:**

The association between periatrial adiposity and atrial arrhythmias has been shown in previous studies. However, there are not enough available data on the association between epicardial fat tissue (EFT) thickness and parameters of ventricular repolarization. Thus, we aimed to evaluate the association of EFT thickness with indices of ventricular repolarization by using T-peak to T-end (Tp-e) interval and Tp-e/QT ratio.

**Methods:**

The present study included 50 patients whose EFT thickness ≥ 9 mm (group 1) and 40 control subjects with EFT thickness < 9 mm (group 2). Transthoracic echocardiographic examination was performed in all participants. QT parameters, Tp-e intervals and Tp-e/QT ratio were measured from the 12-lead electrocardiogram.

**Results:**

QTd (41.1 ± 2.5 vs 38.6 ± 3.2, p < 0.001) and corrected QTd (46.7 ± 4.7 vs 43.7 ± 4, p = 0.002) were significantly higher in group 1 when compared to group 2. The Tp-e interval (76.5 ± 6.3, 70.3 ± 6.8, p < 0.001), cTp-e interval (83.1 ± 4.3 vs. 76±4.9, p < 0.001), Tp-e/QT (0.20 ± 0.02 vs. 0.2 ± 0.02, p < 0.001) and Tp-e/QTc ratios (0.2 ± 0.01 vs. 0.18 ± 0.01, p < 0.001) were increased in group 1 in comparison to group 2. Significant positive correlations were found between EFT thickness and Tp-e interval (r = 0.548, p < 0.001), cTp-e interval (r = 0.259, p = 0.01), and Tp-e/QT (r = 0.662, p < 0.001) and Tp-e/QTc ratios (r = 0.560, p < 0.001).

**Conclusion:**

The present study shows that Tp-e and cTp-e interval, Tp-e/QT and Tp-e/QTc ratios were increased in subjects with increased EFT, which may suggest an increased risk of ventricular arrhythmia.

## Introduction

QT interval (QT), corrected QT interval (QTc), QT dispersion and transmural dispersion of repolarization are generally used for the evaluation of myocardial repolarization. Tp-e, which is the interval between the peak and the end of T wave on electrocardiogram (ECG), is accepted as an index of transmural dispersion of ventricular repolarization^1^. However, it is affected by variations in heart rate and body weight. Tp-e/QT and Tp-e/QTc ratios have been suggested as more accurate measures for the dispersion of ventricular repolarization compared to others parameters, and are independent from heart rate alterations^2,3^.

Growing evidence has recently suggested that epicardial fat tissue (EFT), a particular form of visceral fat deposited around the heart, may be a new marker of visceral adiposity and an important source of inflammatory mediators^4-6^. Furthermore, because of the close anatomic proximity to the heart and the absence of fascial boundaries between EFT and the heart, EFT may locally interact with the coronary arteries and myocardium via production of proinflammatory adipokines, which can enhance local inflammation and directly induce myocardial remodeling^6-9^.

Previous studies have consistently shown an association between EFT and atrial arrhythmia such as atrial fibrillation (AF)^10-12^. However, there are not enough available data regarding the association between EFT and ventricular arrhythmia. Therefore, we aimed to evaluate the possible association between EFT and ventricular repolarization, which is an indicator of ventricular arrhythmia risk.

## Methods

### Study population

Participants were recruited among patients admitted to the cardiology department of our hospital for general control. A total of 90 consecutive subjects were included in the present study. The number of the study participants was based on the power analysis. Patients were divided into two groups. The first group (Group 1) consists of subjects with EFT thickness ≥ 9 mm and the second group (Group 2) consists of subjects with EFT thickness < 9 mm. EFT thickness was chosen according to previous studies^13-16^. All patients’ baseline information including age, gender and body mass index (BMI) was recorded and cardiovascular risk factors were determined: hypertension (HT), diabetes mellitus, smoking and cardiovascular medication use [angiotensin-converting enzyme inhibitors (ACEI), angiotensin-II receptor blockers (ARB), calcium-channel blockers (CCB), β-blockers, antiarrhythmic agents and statins]. Patients with a documented history of coronary artery disease by coronary angiography or computed tomography angiography, moderate-to-severe valvular heart disease, prior pacemaker implantation, AF, heart failure, chronic lung disease, cerebrovascular disease, hepatic or renal failure (alanine aminotransferase and aspartate aminotransferase > 2-fold normal levels, serum creatinine > 1.5 mg/dL), bundle branch block and atrioventricular conduction abnormalities on ECG, abnormal thyroid function test, abnormal electrolyte values, use of β-blockers, CCBs and antiarrhythmic agents were excluded from the study. ECGs without clearly analyzable Tp-e interval and QT segment were also excluded. All patients were in sinus rhythm and none of them were taking medications affecting QT and Tp-e intervals such as antibiotics, tricyclic antidepressants, antihistaminics and antipsychotics. The study was approved by the local ethics committees and adhered to the Declaration of Helsinki, and all subjects gave written informed consent.

### Echocardiographic and Electrocardiographic Examination

**Figure 1:**
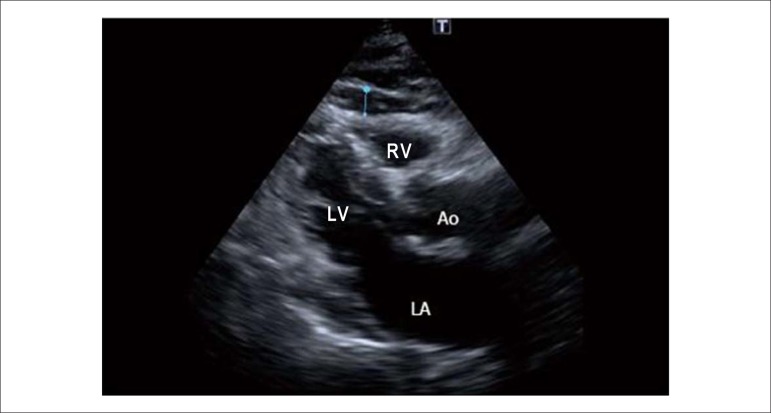
Measurement of epicardial fat thickness by two-dimensional transthoracic echocardiography. RV: Right ventricle; LV: Left ventricle; Ao: Aorta; LA: Left atrium.

All echocardiographic examinations (Vivid 7 Pro, GE Vingmed, Milwaukee, Wisconsin, USA) were performed in all patients with the 4-Mhz transducer of Vivid 7 pro (GE Vingmed, Milwaukee, Wisconsin, USA). Interpretation of echocardiographic examinations was performed by two cardiologists who were blinded to ECG measurements of the study population. During echocardiographic examination, 1-lead ECG was recorded continuously, and three consecutive cycles were averaged for every measured parameter. Two-dimensional and pulsed Doppler measurements were performed according to the criteria of the American Society of Echocardiography ^17^. The following two-dimensional echocardiographic parameters were measured: left ventricular end-diastolic diameter (LVEDD, mm), left ventricular end-systolic diameter (LVESD, mm), left ventricular ejection fraction (LVEF, %), left atrium (LA) and EFT. The LVEF was estimated using Simpson’s rule. The EFT was measured according to a previously described and validated method^6^. Briefly, the epicardial fat was identified as the echo-free space between the myocardium outer wall and the pericardium visceral layer and it was measured perpendicularly on the free wall of the right ventricle at the end diastole in the transthoracic parasternal long-axis view in three cardiac cycles (Figure 1). The maximum value at any site was measured and the average of 3 values was calculated.

**Figure 2:**
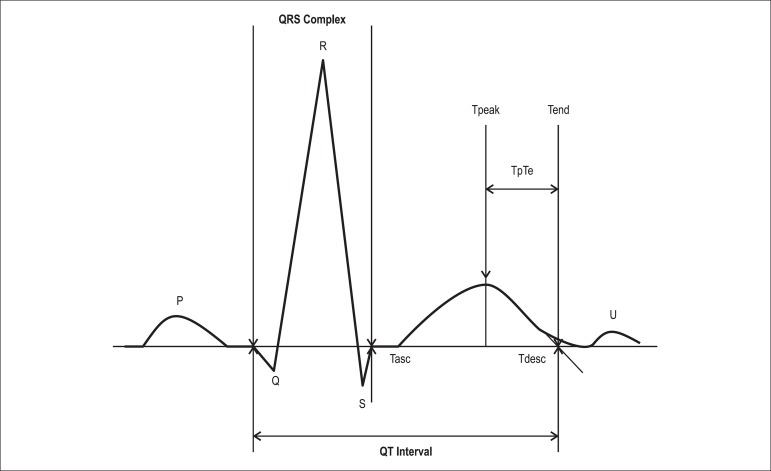
Electrocardiographic parameters measured when assessing the QT interval and Tp-e interval.

The 12-lead ECG was performed at a paper speed of 50 mm/s with the subject at rest in the supine position. The resting heart rate was then measured from the ECG data. ECG measurements of QT and Tp-e intervals were performed manually by two different cardiologists, using calipers and a magnifying glass to decrease measurement errors. The cardiologists were blinded to the echocardiographic measurements of the study population. Subjects with U waves on their ECGs were excluded from the study. The average value of three examinations was calculated for each lead. The QT interval was measured from the beginning of the QRS complex to the end of the T wave, and corrected for heart rate using the Bazett formula^18^. The QTd was defined as the difference between the maximum (QTmax) and minimum QT (QTmin) intervals of the 12 leads. The difference between the corrected QTmax (cQTmax) and corrected QTmin (cQTmin) was defined as corrected QTd (cQTd)^19^. The Tp-e was measured in each precordial lead and obtained from the difference between QT interval and QT peak interval; measured from the beginning of the QRS until the peak of the T-wave (Figure 2). In case of negative or biphasic T waves, QT peak was measured to the nadir of the T-wave. T waves smaller than 1.5 mm in amplitude were not measured. The reported Tp-e value was the maximum obtained by two observers in all precordial leads^20^.

### Statistical Analysis

SPSS 17.0 statistical program (SPSS Inc., Chicago, IL, USA) was used for the statistical study. All parametric values were shown as means with standard deviation. Continuous variables were compared between groups using the Student’s *t* test or Mann-Whitney U test, according to whether normally distributed or not, as tested by the Kolmogorov-Smirnov test. The chi-square test was used to assess differences between categorical variables. Pearson’s correlation analysis was used to examine possible associations between EFT and ventricular repolarization parameters. A p value of less than 0.05 was considered significant.

## Results

In all, 102 patients were enrolled in the present study, of which 12 were excluded for reasons such as ECGs without clearly analyzable Tp-e interval and QT segment. Baseline clinical, demographic and echocardiographic parameters of the study participants are listed in Table 1. Age, gender, BMI, smoking status, HT and dyslipidemia were similar between the two groups, as were LVEDD, LVESD, EF, LA diameter, IVS and PW. EFT thickness of Group 1 and 2 were 10.6 ± 1.1 and 6.2 ± 1.0 mm, respectively (p < 0.001).

**Tabela 1:** Características basais, parâmetros laboratoriais e ecocardiográficos da população estudada

**Variable**	**EFT thickness ≥ 9 mm (n = 50)**	**EFT thickness < 9 mm (n = 40)**	**p value**
Age, years	61.6 ± 8.6	62.2 ± 6.4	0.71
Gender, female/male	21/29	19/21	0.60
BMI, kg/m^2^	28.5 ± 2.7	28.1 ± 3	0.51
Dyslipidemia, n (%)	26(52)	21(52)	1.0
Hypertension, n (%)	35(70)	28(70)	1.0
Smokers, n (%)	15(30)	12(30)	1.0
Glucose, mg/dL	88.2 ± 6.4	87.6 ± 6.6	0.64
TC, mg/dL	214.5 ± 17	213 ± 20.3	0.60
Triglyceride, mg/dL	160.2 ± 18.7	162 ± 17.1	0.52
LDL-C, mg/dL	136.2 ± 9.3	135 ± 9.2	0.66
HDL-C, mg/dL	38.5 ± 2.4	38.8 ± 2.4	0.70
Statins, n (%)	13(26)	9(22)	0.70
ACEI/ARB, n (%)	23(65)	14(50)	0.20
CCB, n (%)	13(37)	15(53)	0.19
LVEDD, mm	46.4 ± 1.9	46.8 ± 2.1	0.41
LVESD, mm	29.4 ± 1.8	29.5 ± 1.9	0.75
LA, mm	35.2 ± 2.2	34.8 ± 2.2	0.72
IVS, mm	9.8 ± 0.9	10.0 ± 0.9	0.46
PW, mm	8.8 ± 0.6	8.9 ± 0.6	0,75
LVEF, %	56.1 ± 1.4	55.7 ± 1.2	0.20
EFT thickness, mm	10.6 ± 1.1	6.2 ± 1.0	< 0.001

ACEI: Angiotensin-converting enzyme inhibitor; ARB: Angiotensin receptor blocker; BMI: Body mass index; CCB: Calcium channel blocker; EFT: Epicardial fat tissue; HDL-C: High-density lipoprotein cholesterol; IVS: Interventricular septum; LA: Left atrium; LDL-C: Low-density lipoprotein cholesterol; LVEDD: Left ventricular end-diastolic diameter; LVEF: Left ventricular ejection fraction; LVESD: Left ventricular end-systolic diameter; PW: Posterior wall; TC: Total cholesterol.

The ECG parameters of the groups are shown in Table 2. Heart rate was similar between the two groups. The QTmax (p = 0.06), cQTmax (p = 0.01), QTmin (p = 0.03), cQTmin (p = 0.003), QTd (p < 0.001) and cQTd (p = 0.002) were significantly increased in Group 1 in comparison to Group 2. The Tp-e interval (p < 0.001), cTp-e interval (p < 0.001), Tp-e/QT (p < 0.001) and Tp-e/QTc ratios (p < 0.001) were also increased in Group 1 when compared to Group 2. Significant positive correlations were found between EFT thickness and Tp-e interval (r = 0.548, p < 0.001), cTp-e interval (r = 0.259, p = 0.014), and Tp-e/QT (r = 0.662, p < 0.001) and Tp-e/QTc ratios (r = 0.560, p < 0.001) (Table 3). Reproducibility data for the measurements of EFT thickness, QTmax, QTmin and Tp-e interval in 20 reexamined participants are shown in Table 4.

**Table 2:** Electrocardiographic parameters of the study population

**Variable**	**EFT thickness ≥ 9 mm**	**EFT thickness < 9 mm**	**p value**
HR, (beat/min)	78.2 ± 12.7	78.7 ± 11.5	0.86
QTmax, (ms)	357 ± 36	370 ± 26	0.06
cQTmax, (ms)	404 ± 26	417 ± 26.2	0.01
QTmin, (ms)	316 ± 35	331 ± 27	0.03
cQTmin, (ms)	357 ± 25	374 ± 25	0.003
QTd, (ms)	41.1 ± 2.5	38.6 ± 3.2	< 0.001
cQTd, (ms)	46.7 ± 4.7	43.7 ± 4.0	0.002
Tp-e, (ms)	83.1 ± 4.3	76.0 ± 4.9	< 0.001
cTp-e, (ms)	95.1 ± 12.0	86.5 ± 8.0	< 0.001
Tp-e/QT	0.23 ± 0.02	0.20 ± 0.02	< 0.001
Tp-e/QTc	0.20 ± 0.01	0.18 ± 0.01	< 0.001

HR: Heart rate; QTmax: QTmaximum; cQTmax: Corrected QT maximum; QTmin: QT minimum; cQTmin: Corrected QT minimum; QTd: QT dispersion; cQTd: CorrectedQT dispersion; Tp-e: Transmural dispersion of repolarization; cTp-e: Corrected transmural dispersion of repolarization; EFT: Epicardial fat tissue.

**Table 3:** Correlations between EFT and electrocardiographic parameters

**Variable**	**EFT thickness**	
	**R**	**p**
Tp-e interval	0.548	< 0.001
cTp-e interval	0.259	0.014
Tp-e/QT	0.662	< 0.001
Tp-e/QTc	0.560	< 0.001

QTc: Corrected QT; EFT: Epicardial fat tissue; Tp-e: Transmural dispersion of repolarization; cTp-e: Corrected transmural dispersion of repolarization.

**Table 4:** Reproducibility data for the measurements of echocardiographic and electrocardiographic parameters

	**Intraobserver (%)**	**Interobserver (%)**
EFT thickness	6.7	8.5
QTmax	2.8	3.2
QTmin	2.8	3.1
Tp-e interval	2.8	3.1

EFT: Epicardial fat tissue. QTmax: QTmaximum; QTmin: QT minimum; Tp-e: Transmural dispersion of repolarization.

## Discussion

We found that the Tp-e and cTp-e intervals, the Tp-e/QT and Tp-e/QTc ratios, were higher in patient with increased EFT thickness compared with controls. These ECG parameters of ventricular repolarization were also significantly correlated with the EFT thickness. Our finding of increased Tp-e, cTp-e, Tp-e/QT ratio, and Tp-e/QTc ratio in patients with increased EFT is important, as this is the first study evaluating the association between EFT thickness and parameters of ventricular repolarization. Our results may contribute to the knowledge of the pathophysiological mechanisms of increased prevalence of ventricular arrhythmias in patients with higher EFT thickness.

Different echocardiographic studies have adopted different cut-off values for increased EFT. Iacobellis et al showed that EFT values were increased when > 9.5 mm in men and 7.5 mm in women with metabolic syndrome^13^. In addition, the authors adopted EFT values as elevated when > 9.5 mm in men and above 9.5 mm in women with insulin resistance. Natale et al^14^ accepted cut-off values for increased EFT as those higher than 7 mm in men and higher than 7 mm in women with subclinical atherosclerosis. Eroglu et al^15^ adopted different cut-off values for increased EFT, as > 5.2 mm in men and > 5.2 mm in women with coronary artery disease. Pierdomenico et al^16^ disclosed that EFT values were 2.5-7.1 mm in the normal population in a meta-analysis. All study populations have European ethnicity. We established a cutoff value of 9 mm for increased EFT.

EFT has a smaller adipocyte size but higher rates of fatty acid uptake and secretion than other visceral fat depots^21,22^. However, epicardial fat has some vital benefits, such as serving as a buffer, absorbing fatty acids, and protecting the heart against high fatty acid levels. In addition, it is used as a local energy source at times of high demand by channeling fatty acids to the myocardium^22^. In fact, the body of evidence shows that epicardial fat is an extremely active organ that secretes several activated pro-inflammatory cytokines, such as tumor necrosis factor-α, transforming growth factor-β (TGF-β), and interleukin-6 (IL-6)^4,22^. Furthermore, because of its proximity to the heart and its shared blood supply with the coronary arteries, EFT may induce electrical and structural remodeling of the heart, leading to ventricular arrhythmias. In previous studies, it was shown that epicardial fat was associated to heart failure, coronary heart disease, metabolic syndrome, HT and AF^10-12,23-26^.

A recent study demonstrated that pericardial fat volume was highly associated with paroxysmal and persistent AF regardless of traditional risk factors, including LA enlargement^27^. Furthermore, the Framingham heart study revealed that pericardial fat, but not other fat deposits, was associated with prevalent AF^28^. According to these previous studies we thought that there was an association between EFT and dysrhythmia and then we hypothesized that local interactions between EFT and the adjacent myocardium might cause structural remodeling and, consequently, contribute to the genesis of ventricular arrhythmias. These results suggest that the increase in regional epicardial fat might play an important role in structural remodeling. Although the mechanism underlying the association between increased EFT thickness and ventricular arrhythmias is uncertain, the present data may imply that EFT may contribute to the progression of ventricular remodeling.

After several studies showed an association between prolonged Tp-e interval and ventricular arrhythmogenesis and sudden cardiac death, this parameter has gained great popularity^3,20^. In addition, the Tp-e/QT ratio is considered to be a more sensitive index of arrhythmogenesis compared with the sole use of either the Tp-e or QT intervals, as it is not affected by variations in body weight and heart rate^2^. Furthermore, electrophysiological studies showed that a prolonged Tp-e interval was correlated with ventricular tachycardia (VT) induction and the spontaneous occurrence of VT^29,30^. Moreover, a higher Tp-e/QT ratio has been associated with arrhythmic events in many clinical conditions, such as Brugada syndrome, long-QT syndromes, hypertrophic cardiomyopathy, and undergoing primary percutaneous coronary intervention for myocardial infarction^2^.

### Study limitations

We recognize that our study has limitations that warrant consideration. First, the observational and cross-sectional design does not allow us to infer causation between EFT thickness and ECG parameters. Second, the sample size of the study was relatively small and follow-up was not long enough to detect any ventricular arrhythmias in patients with higher EFT thickness. Thirdly, the cut-off values of EFT thickness between study groups are taken arbitrarily, which may affect statistical results. Nevertheless, our cut-off value of 9 mm was higher than that seen in previous studies assessing EFT in various disease groups ^16,23-26^. Lastly, this study may provide knowledge that can be used in large prospective studies.

## Conclusion

Tp-e interval, and Tp-e/QT, and Tp-e/QTc ratios were elevated in patients with higher EFT thicknesses, which might imply an indicator of risk of ventricular arrhythmias in this group of patients.

Institution name and approval number: istanbul bilim university-2014/176.*
